# Complete plastome sequencing from *Toona* (Meliaceae) and phylogenomic analyses within Sapindales

**DOI:** 10.1002/aps3.1040

**Published:** 2018-04-27

**Authors:** Nan Lin, Michael J. Moore, Tao Deng, Hang Sun, Lin‐sen Yang, Yan‐xia Sun, Heng‐chang Wang

**Affiliations:** ^1^ Key Laboratory of Plant Germplasm Enhancement and Specialty Agriculture Wuhan Botanical Garden Chinese Academy of Sciences Wuhan Hubei China; ^2^ University of the Chinese Academy of Sciences Beijing China; ^3^ Key Laboratory for Plant Diversity and Biogeography of East Asia Kunming Institute of Botany Chinese Academy of Sciences Kunming Yunnan China; ^4^ Department of Biology Oberlin College Oberlin Ohio USA; ^5^ Hubei Key Laboratory of Shennongjia Golden Monkey Conservation Biology Administration of Shennongjia National Park Shennongjia Hubei China

**Keywords:** phylogenomic analysis, plastome, Sapindales, structure, *Toona*

## Abstract

**Premise of the Study:**

*Toona* (Meliaceae, Sapindales) is a small genus of five species of trees native from southern and eastern Asia to New Guinea and Australia. Complete plastomes were sequenced for three *Toona* species to provide a basis for future plastome genetic studies in threatened species of *Toona*. In addition, plastome structural evolution and phylogenetic relationships across Sapindales were explored with a larger data set of 29 Sapindales plastomes (including members of six out of nine families).

**Methods:**

The plastomes were determined using the Illumina sequencing platform; the phylogenetic analyses were conducted using maximum likelihood by RAxML.

**Results:**

The lengths of three *Toona* plastomes range from 159,185 to 158,196 bp. A total of 113 unique genes were found in each plastome. Across Sapindales, plastome gene structure and content were largely conserved, with the exception of the contraction of the inverted repeat region to exclude *ycf1* in some species of Rutaceae and Sapindaceae, and the movement of *trnI‐GAU* and *trnA‐UGC* to a position outside the inverted repeat region in some Rutaceae species.

**Discussion:**

The three *Toona* plastomes possess the typical structure of angiosperm plastomes. Phylogenomic analysis of Sapindales recovered a mostly strongly supported phylogeny of Sapindales, including most of the backbone relationships, with some improvements compared to previous targeted‐gene analyses.

*Toona* (Endl.) M. Roem., commonly known as red cedar, is a small genus of trees in the mahogany family (Meliaceae subfam. Cedreloideae). It is distributed across southern and eastern Asia, New Guinea, and eastern Australia (Mabberley, 2008). *Toona* was previously treated as a section of *Cedrela* P. Browne (Meliaceae), but the latter is now circumscribed to include only species of the Neotropics (Muellner et al., 2009). Approximately five species of *Toona* are currently recognized following the treatment by J. M. Edmonds (1995): *T. calantas* Merr. & Rolfe, *T. ciliata* M. Roem., *T. fargesii* A. Chev., *T. sinensis* (A. Juss.) M. Roem., and *T. sureni* (Blume) Merr. (Fig. 1). Several of these species are economically important as timber trees (e.g., *T. ciliata* and *T. sureni*; Peng and Edmonds, 2008) or as ornamental, including *T. sinensis*, which is the most cold‐tolerant species in Meliaceae and the only member of the family that can be cultivated successfully in northern Europe (Rushforth, 1999). Wild populations of most *Toona* species are under threat due to habitat loss and logging, especially the extremely rare *T. fargesii*, which may be endemic to China (Peng and Edmonds, 2008).

**Figure 1:**
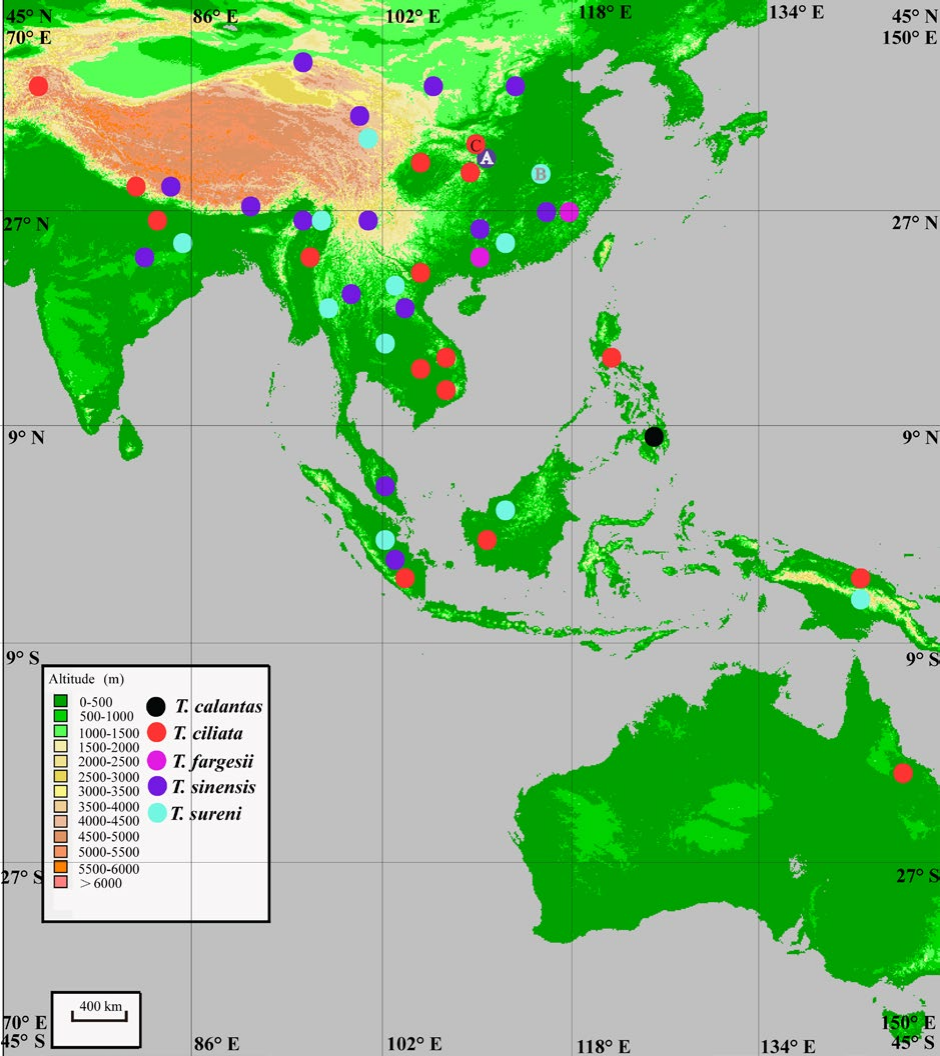
The distribution pattern of *Toona*. The colored dots represent the species range. A, B, and C indicate the sampling localities of three *Toona* species sequenced in the present study.

The large pantropical family Meliaceae is a member of the order Sapindales (Angiosperm Phylogeny Group, 2016) and consists of 50 genera and more than 650 species (Stevens, 2001 onwards). Meliaceae is strongly supported as monophyletic and consists of two subfamilies: Cedreloideae and Melioideae (Muellner et al., 2003). A recent phylogenetic study of Sapindales based on plastid *rbcL*,* atpB*, and *trnL*‐*trnF* sequences (Muellner‐Riehl et al., 2016) found that Simaroubaceae was sister to Meliaceae, with moderate support. Together, these two families formed a strongly supported clade with Rutaceae. Relationships among the remaining families of Sapindales were mostly moderately to strongly supported. Resolution and support found in Muellner‐Riehl et al. (2016) represent improvements over earlier studies based on fewer loci (e.g., Gadek et al., 1996; Muellner et al., 2007).

Phylogenetic data sets based on large numbers of plastid loci have the potential to resolve relationships that have resisted resolution using only a few loci, as has been demonstrated in many recent studies (e.g., Stull et al., 2015; Duvall et al., 2016). Plastomes are generally conserved in structure, gene content, and gene order (Green, 2011; Ruhlman and Jansen, 2014), although rearrangements and gene loss have been detected in a number of lineages and most differences in plastome gene number are related to fluctuations in the size of the inverted repeat (IR) region (e.g., Guisinger et al., 2011; Knox, 2014; Zhu et al., 2016). To date, complete plastomes of 26 species across six families are available for Sapindales, including one Meliaceae species (*Azadirachta indica* A. Juss., Melioideae). Although McPherson et al. (2013) sequenced the *T. ciliata* plastome for phylogeographical study of this species in Australia, the plastome structure of this species was not reported, and the assembled plastome sequences of this species are not openly available. Additional sequenced plastomes from Meliaceae as well as across Sapindales may help to improve our understanding of phylogenetic relationships within the order and would provide insight into plastome evolution in this clade. In this study, we sequenced and characterized the complete plastomes of three *Toona* species and downloaded all 26 available Sapindales plastomes from GenBank, with the following objectives: (1) to provide a basis for future plastome genetic studies in threatened species of *Toona*, (2) to determine whether plastomes can resolve phylogenetic relationships among families of Sapindales, and (3) to evaluate plastome structure evolution across Sapindales.

## METHODS

Fresh leaves of *T. sinensis*,* T. sureni*, and *T. ciliata* were obtained from Wuhan Botanical Garden (30.54°N, 110.42°E), Lushan Botanical Garden (29.55°N, 115.99°E), and the National Nature Reserve of Shi‐Ba‐Li valley (31.34°N, 109.92°E), respectively. Vouchers were deposited at the Herbarium of Wuhan Botanical Garden, Chinese Academy of Sciences (HIB) (Table 1). High‐quality plastid DNA was obtained following the plastid DNA extraction method of Shi et al. (2012). Approximately 30 g of fresh, young leaf tissue was used for each species, and for each plastome a DNA TruSeq Illumina (Illumina Inc., San Diego, California, USA) sequencing library, with 500‐bp insert sizes, was constructed at the Beijing Genomics Institute (BGI) in Wuhan, Hubei, China, using 2.5–5 ng of sonicated plastid DNA. An Agilent 2100 Bioanalyzer (Agilent Technologies, Santa Clara, California, USA) and quantitative PCR were used to quantify DNA amounts in the libraries. Libraries were multiplexed by TruSeq adapter and 150‐bp paired‐end sequenced on an Illumina HiSeq 2000 platform at BGI (Wuhan, Hubei, China). The raw data are available from the National Center for Biotechnology Information Sequence Read Archive (accession no. SRR6146642, SRR6146640, and SRR6146641).

**Table 1:** Taxa used in present study. Collection locality and voucher information are provided for newly sequenced plastomes

Family	Species	Collection locality	Voucher information	GenBank accession no.
Anacardiaceae	*Rhus chinensis* Mill.	Yanggu, Korea	IM151120‐1 (Lee et al., 2016)	NC_033535
Anacardiaceae	*Spondias bahiensis* P. Carvalho, Van den Berg & Machado	NA	NA	NC_030526
Anacardiaceae	*Spondias tuberosa* L.	NA	NA	NC_030527
Burseraceae	*Boswellia sacra* Flueck.	Natural Park	UC29 (Kohany et al., 2006)	NC_029420
Meliaceae	*Azadirachta indica* A. Juss.	NA	NA	NC_023792
Meliaceae	*Toona ciliata* M. Roem.	SBL	Nan.Lin‐521(HIB)	MF467523
Meliaceae	*Toona sinensis* (A. Juss.) M. Roem.	WBG	Nan.Lin‐522 (HIB)	MF467522
Meliaceae	*Toona sureni* (Blume) Merr.	LBG	Nan.Lin‐523 (HIB)	MF467521
Rutaceae	*Citrus aurantiifolia* (Christm.) Swingle	Omani, Madha	Su et al., 2014	KJ_865401
Rutaceae	*Citrus depressa* Hayata	Okinawa, Japan	Ishikawa et al., 2016	LC147381
Rutaceae	*Citrus platymamma* Tanaka	Jeju Island, Korea	Lee et al., 2015	NC_030194
Rutaceae	*Citrus sinensis* (L.) Osbeck	USA	Bausher et al., 2006	NC_008334
Rutaceae	*Clausena excavata* Burm. f.	USDA	PI539715 (Shivakumar et al., 2016)	NC_032685
Rutaceae	*Glycosmis mauritiana* (Lam.) Tanaka	USDA	PI600641 (Shivakumar et al., 2016)	KU949004
Rutaceae	*Glycosmis pentaphylla* (Retz.) DC.	USDA	PI127866 (Shivakumar et al., 2016)	NC_032687
Rutaceae	*Merrillia caloxylon* (Ridl.) Swingle	USDA	PI539733 (Shivakumar et al., 2016)	NC_032688
Rutaceae	*Micromelum minutum* Wight & Arn.	USDA	PI539744 (Shivakumar et al., 2016)	NC_032689
Rutaceae	*Murraya koenigii* (L.) Spreng.	USDA	PI539745 (Shivakumar et al., 2016)	NC_032684
Rutaceae	*Zanthoxylum bungeanum* Maxim.	Fengxian, China	Liu and Wei, 2017	KX497031
Rutaceae	*Zanthoxylum piperitum* DC.	NA	Lee et al., 2015	NC_027939
Rutaceae	*Zanthoxylum schinifolium* Siebold & Zucc.	NA	IM2014_ZS (Lee et al., 2016)	NC_030702
Sapindaceae	*Acer buergerianum* Miq.	NA	Sd0060 (Yang et al., 2014)	KF753631
Sapindaceae	*Acer davidii* Franch.	Changan, China	EBL (Jia et al., 2016)	NC_030331
Sapindaceae	*Acer miaotaiense* P. C. Tsoong	Shaanxi, China	MTQ20160406SAXHZ (Zhang et al., 2016)	NC_030343
Sapindaceae	*Acer morrisonense* Hayata	Shaanxi, China	Amorr2015 (Li et al., 2017)	NC_029371
Sapindaceae	*Dipteronia dyeriana* A. Henry	Shaanxi, China	Zhou et al., 2016	NC_031899
Sapindaceae	*Dipteronia sinensis* Oliv.	Shaanxi, China	Zhou et al., 2016	NC_029338
Sapindaceae	*Sapindus mukorossi* Gaertn.	NA	Yang et al., 2016	NC_025554
Simaroubaceae	*Leitneria floridana* Chapm.	NA	MO:MO 2008‐0670 (Yang et al., 2014)	NC_030482

Note

HIB = Herbarium of Wuhan Botanical Garden, Chinese Academy of Sciences; LBG = Lushan Botanical Garden, Jiangxi, China; NA = not available; SBL = National Nature Reserve of Shi‐Ba‐Li valley, Shiyan, China; WBG = Wuhan Botanical Garden, Wuhan, China; USDA = United States Department of Agriculture.

The raw reads were subsequently filtered for high‐quality reads following the method described by Sun et al. (2016). Filtered reads were assembled into contigs with a minimum length of 1000 bp using CLC Genomics Workbench 9 (Girard et al., 2011) with default parameters, except that the *k*‐mer value was set to 60 for *T. sinensis* and *T. sureni*, and 64 for *T. ciliata*, to produce the highest N50 value. The assembly statistics are presented in Appendix 1. After trimming, the contigs were ordered according to the reference genome *Azadirachta indica* A. Juss. (NC_023792). Plastid genomes were annotated with DOGMA (Wyman et al., 2004), and gene start and stop codons were determined through comparison to start and stop codons in the homologous genes of *A. indica*. Annotation of tRNA genes was conducted using tRNAscan‐SE (Schattner et al., 2005). Junctions between large single‐copy regions (LSCs) and IRs and small single‐copy regions (SSCs) and IRs of the three plastomes were verified with PCR and Sanger sequencing. Physical maps of plastomes were generated using GenomeVx (Conant and Wolfe, 2008).

In total, 79 protein‐coding regions and the *ycf15* region were identified from the plastomes of three *Toona* species and 26 other species of Sapindales, with two taxa of Malvales (*Cytinus hypocistis* (L.) L. and *Hibiscus syriacus* L.) as outgroups (Table 1). These sequences were then manually compiled into a single file of the 31‐taxon data set and aligned with MAFFT (Katoh et al., 2002) for phylogenetic analyses. GenBank information for all plastomes used for phylogenetic analyses are provided in Table 1. In order to further investigate the phylogenetic relationships within Sapindales, maximum likelihood (ML) analyses were conducted using RAxML version 7.4.2 (Stamatakis et al., 2008) under the general time‐reversible (GTR) substitution model. We conducted both unpartitioned and partitioned analyses. PartitionFinder version 1.1.1 (Lanfear et al., 2012) was employed to determine the best‐fit partition scheme for partitioned ML analysis. Bootstrap support was estimated with 1000 bootstrap replicates.

In order to be convenient for subsequent population genetic study within *Toona*, simple sequence repeats (SSRs) were detected using MISA (Thiel et al., 2003) with thresholds of 10 repeat units for mononucleotide SSRs, five repeat units for di‐ and trinucleotide SSRs, and three repeat units for tetra‐, penta‐, and hexanucleotide SSRs. Additionally, repeat sequences were identified for each plastome using REPuter (Kurtz et al., 2001) with a minimum repeat size of 30 bp. Single‐nucleotide polymorphisms (SNPs) and insertion/deletion polymorphisms (indels) were also identified among three *Toona* plastomes with Geneious 7.0 (Kearse et al., 2012).

## RESULTS

**Figure 2:**
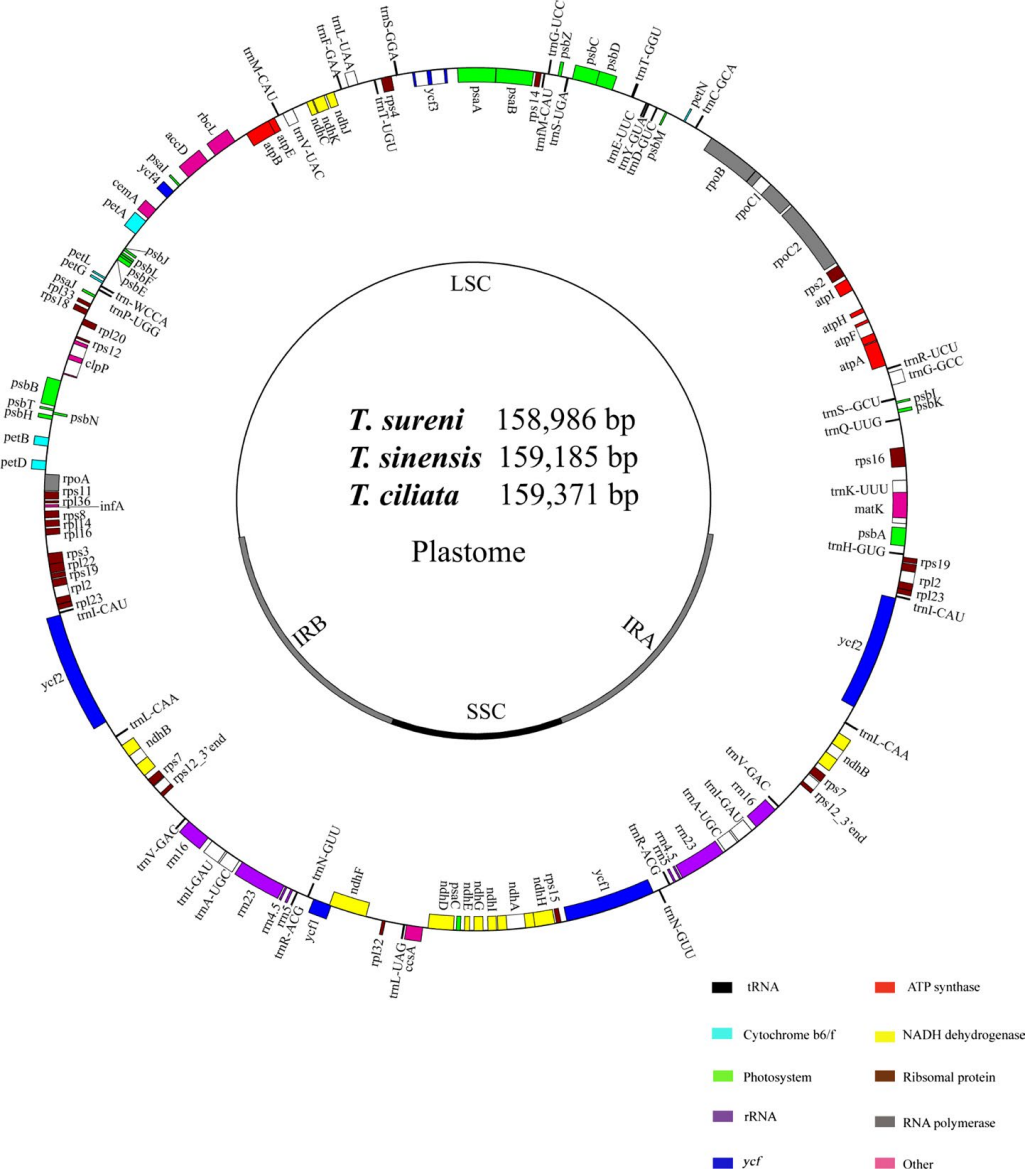
Physical maps of three *Toona* plastomes.

Within *Toona*, the plastome size of *T. sureni* was 159,371 bp, and those of *T. sinensis* and *T. ciliata* were 186 bp and 385 bp longer, respectively (Table 2). These three plastomes possess the typical quadripartite structure of angiosperm plastomes, comprising an LSC, an SSC, and two IR regions (Fig. 2). A total of 113 unique genes, including 30 tRNA genes, four rRNA genes, and 79 protein‐coding genes were found in each plastome. Nineteen genes were duplicated in the IR regions (Table 3). Additionally, 14 genes were found to possess one intron, and three genes (*rps12*,* clpP*,* ycf3*) were found to possess two introns (Appendix 2).

**Table 2:** Plastome characteristics of Sapindales included in this study. Three *Toona* species were sequenced for the first time in this study, and other species were accessed from the National Center for Biotechnology Information database

Family	Species	Total genome length (bp)	LSC length (bp)	SSC length (bp)	IR length (bp)	No. of genes within IR	Overall G/C content (%)
Anacardiaceae	*Rhus chinensis*	149,011	96,882	18,647	16,741	18	37.8
Anacardiaceae	*Spondias bahiensis*	162,218	89,606	18,382	27,075	19	37.7
Anacardiaceae	*Spondias tuberosa*	162,039	89,453	18,368	27,139	19	37.7
Burseraceae	*Boswellia sacra*	160,543	88,054	18,962	26,764	20	37.6
Meliaceae	*Azadirachta indica*	160,737	88,137	18,624	26,983	19	37.5
Meliaceae	*Toona ciliata*	158,986	87,163	18,329	26,747	19	37.9
Meliaceae	*Toona sinensis*	159,185	87,358	17,933	26,947	19	37.9
Meliaceae	*Toona sureni*	159,371	87,505	18,472	26,697	19	37.9
Rutaceae	*Citrus aurantiifolia*	159,893	87,148	18,762	26,991	20	38.4
Rutaceae	*Citrus depressa*	160,120	87,794	18,376	26,955	20	38.5
Rutaceae	*Citrus platymamma*	160,121	87,732	18,393	26,998	20	38.5
Rutaceae	*Citrus sinensis*	160129	87,744	18,393	26,996	20	38.5
Rutaceae	*Clausena excavata*	161,172	88,055	18,295	27,411	17	38.3
Rutaceae	*Glycosmis mauritiana*	160,131	87,710	18,383	27,019	16	38.5
Rutaceae	*Glycosmis pentaphylla*	159,845	87,494	18,329	27,011	16	38.4
Rutaceae	*Merrillia caloxylon*	159,969	87,912	18,029	27,014	16	38.5
Rutaceae	*Micromelum minutum*	160,416	87,367	18,622	27,214	17	38.5
Rutaceae	*Murraya koenigii*	159,402	87,077	18,123	27,101	16	38.5
Rutaceae	*Zanthoxylum bungeanum*	158,401	85,898	17,611	27,446	19	38.5
Rutaceae	*Zanthoxylum piperitum*	158,154	85,340	17,526	27,644	19	38.5
Rutaceae	*Zanthoxylum schinifolium*	158,963	86,528	18,256	27,089	19	38.4
Sapindaceae	*Acer buergerianum*	156,911	85,314	18,093	26,752	18	37.9
Sapindaceae	*Acer davidii*	157,044	85,410	18,112	26,761	18	37.9
Sapindaceae	*Acer miaotaiense*	156,595	86,327	18,068	26,100	18	37.9
Sapindaceae	*Acer morrisonense*	157,197	85,655	18,086	26,728	18	37.8
Sapindaceae	*Dipteronia dyeriana*	157,071	85,529	18,082	26,730	19	38.0
Sapindaceae	*Dipteronia sinensis*	157,080	85,455	18,093	26,766	19	37.8
Sapindaceae	*Sapindus mukorossi*	160,481	85,649	18,874	27,979	21	37.7
Simaroubaceae	*Leitneria floridana*	158,763	85,689	18,186	27,444	20	37.6

Note

IR = inverted repeat; LSC = large single copy; SSC = small single copy.

**Table 3:** List of genes present in the plastomes of the three *Toona* species

Function	Gene group	Gene name
Protein synthesis and DNA replication	Ribosomal RNAs	*rrn4.5* (×2), *rrn5* (×2), *rrn16* (×2) c *rrn23* (×2)
	Transfer RNAs	*trnH‐GUG*,* trnK‐UUU* a, *trnQ‐UUG*,* trnS‐GCU*,* trnG‐UCC* a, *trnR‐UCU*,* trnC‐GCA*,* trnD‐GUC*,* trnY‐GUA*,* trnE‐UUC*,* trnT‐GGU*,* trnS‐UGA*,* trnG‐UCC*,* trnfM‐CAU*,* trnS‐GGA*,* trnT‐UGU*,* trnL‐UAA* a, *trnF‐GAA*,* trnV‐UAC* a, *trnM‐CAU*,* trnW‐CCA*,* trnP‐UGG*,* trnI‐CAU* a (×2), *trnL‐CAA* (×2), *trnV‐GAC* (×2), *trnI‐GAU* (×2), *trnA‐UGC* a (×2), *trnR‐ACG* (×2), *trnN‐GUU* (×2), *trnL‐UAG*
	Small subunit	*rps2*,* rps3*,* rps4*,* rps7* (×2), *rps8*,* rps11*,* rps12* a (×2), *rps14*,* rps15*,* rps16*,* rps18*,* rps19*
	Ribosomal protein large subunit	*rpl2* a (×2), *rpl14*,* rpl16*,* rpl20*,* rpl22*,* rpl23* (×2), *rpl32*,* rpl33*,* rpl36*
	RNA polymerase	*rpoA*,* rpoB*,* rpoC1* a, *rpoC2*
Photosynthesis	Photosystem I	*psaA*,* psaB*,* psaC*,* psaI*,* psaJ*
	Photosystem II	*psbA*,* psbB*,* psbC*,* psbD*,* psbE*,* psbF*,* psbH*,* psbI*,* psbJ*,* psbK*,* psbL*,* psbM*,* psbN*,* psbT*,* psbZ*
	Cytochrome *b*_6_/*f*	*petA*,* petB*,* petD*,* petG*,* petL*,* petN*
	ATP synthase	*atpA*,* atpB*,* atpE*,* atpF* a, *atpH*,* atpI*
	NADH dehydrogenase	*ndhA* a, *ndhB* a (×2), *ndhC*,* ndhD*,* ndhE*,* ndhF*,* ndhG*,* ndhH*,* ndhI*,* ndhJ*,* ndhK*
	Large subunit of RuBisCO	*rbcL*
Miscellaneous proteins	Subunit of acetyl‐CoA‐carboxylase	*accD*
	c‐type cytochrome synthesis gene	*ccsA*
	Envelope membrane protein	*cemA*
	Protease	*clpP* a
	Translational initiation factor	*infA*
	Maturase	*matK*
Genes of unknown function	Hypothetical conserved coding frame	*ycf1*,* ycf2* (×2), *ycf3* a, *ycf4*

a Genes with introns.

**Figure 3:**
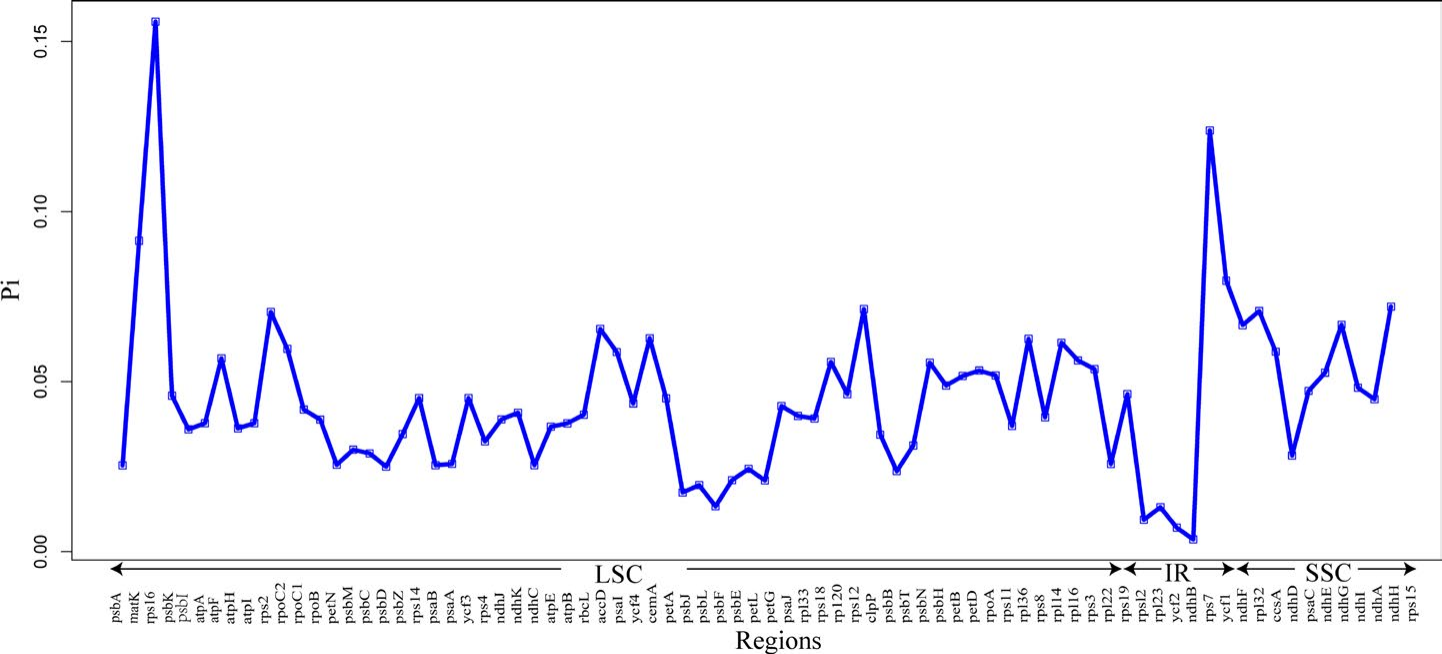
Plot of nucleotide variability (Pi) values among 29 Sapindales plastomes.

Across Sapindales, *Spondias bahiensis* P. Carvalho, Van den Berg & Machado (Anacardiaceae) and *Rhus chinensis* Mill. (Anacardiaceae) possessed the largest (162,218 bp) and smallest (149,011 bp) plastomes, respectively (Table 2). The latter also possessed the longest LSC and the shortest IR regions. *Boswellia sacra* Flueck. (Burseraceae) and *Sapindus mukorossi* Gaertn. (Sapindaceae) possessed the longest SSC and IR regions, respectively. Almost all 29 Sapindales plastomes contained 19 to 20 genes. *Sapindus mukorossi* of Sapindaceae possessed the longest IR region (21 genes). Among all 29 Sapindales plastomes, eight exhibited an IR expansion to *rpl22* at the IR/LSC region boundaries and the IR region of *S. mukorossi* extended to *rps3*. In some Rutaceae (e.g., *Clausena excavata* Burm. f., *Glycosmis mauritiana* (Lam.) Tanaka, *Glycosmis pentaphylla* (Retz.) DC., *Murraya koenigii* (L.) Spreng., *Merrillia caloxylon* (Ridl.) Swingle, and *Micromelum minutum* Wight & Arn.) and Sapindaceae (e.g., *Acer davidii* Franch., *A. morrisonense* Hayata), the IR region was found to have contracted such that all of *ycf1* is now within the SSC region. Moreover, in all of the above‐mentioned six Rutaceae plastomes, both *trnI‐GAU* and *trnA‐UGC* were present in the SSC region, while all rRNA genes were still located in the IR region. In Sapindales, *infA* was found as a pseudogene in several cases of Sapindaceae (e.g., *B. sacra*,* A. davidii*,* A. morrisonense*, and *A. miaotaiense* P. C. Tsoong). The G/C content of all plastomes was approximately 38% among 29 Sapindales plastomes (Table 2). The sequence divergence of 79 protein‐coding genes among all 29 genomes varied from 0.00361 (*rps7*) to 0.1582 (*rps16*). The genes *rps16*,* ycf1*, and *matK* had the highest sequence divergence (0.15582, 0.12381, and 0.09137, respectively; Fig. 3). Notably, *rpl22* was found to have a high variation in length, from 171 bp (*Micromelum minutum*, Rutaceae) to 514 bp (*Toona sureni*, Meliaceae) (Appendix S1).

**Figure 4:**
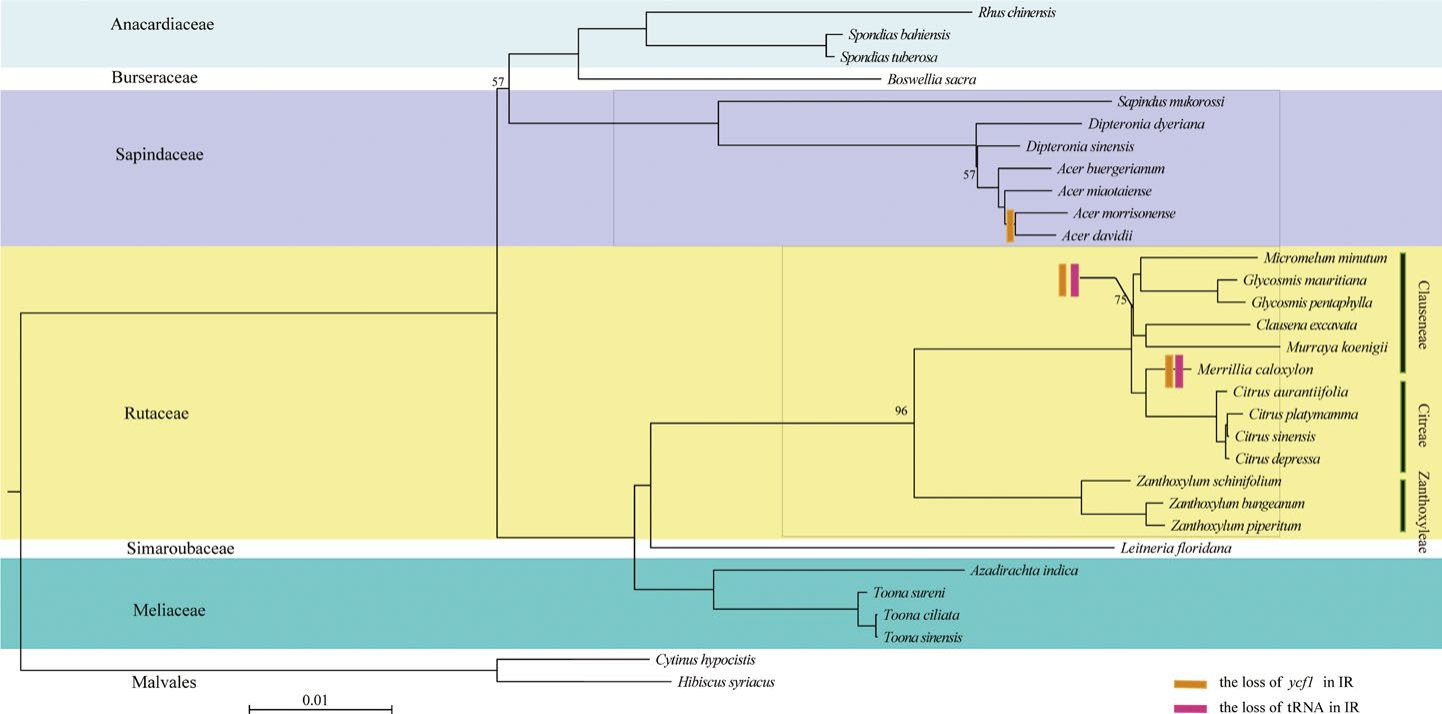
The best maximum likelihood tree of Sapindales based on the 17‐partition analysis of 79 plastid genes (and the *ycf15* region). Numbers above branches are maximum likelihood bootstrap support values (unlabeled branches have bootstrap support of 100%).

The alignment of the 31‐taxon data set was 63,597 bp in length. The best partition scheme determined by PartitionFinder contained 17 partitions (maximum likelihood score [ln *L*] = −229027.17027, Bayesian information criterion [BIC] = 460434.027954). The unpartitioned and partitioned ML analyses yielded identical tree topology, with slightly higher support values in the partitioned tree (Fig. 4; the unpartitioned tree is not shown). Most nodes had very high bootstrap support (Fig. 4), and Anacardiaceae, Sapindaceae, Rutaceae, and Meliaceae were recovered as monophyletic. The backbone of Sapindales was strongly supported except for one node that united Burseraceae, Rutaceae, and Sapindaceae (57%; Fig. 4). Meliaceae was sister to Simaroubaceae + Rutaceae.

A total of 193 SSRs were identified in the three plastomes of *Toona*. Among these, 70 were distributed in *T. sureni*, 57 in *T. sinensis*, and 66 in *T. ciliata* (Appendix 3). The majority of SSRs were A/T mononucleotides, a total of 14 AT dinucleotide repeats were found in the three plastomes, and one TA dinucleotide repeat was detected in *T. sinensis*, whereas the only AG dinucleotide repeat from *T. sureni* was located in the *rpoB‐trnC‐GCA* intergenic region. The other kinds of repeat units (e.g., six dinucleotide; four trinucleotide; three tetra‐, penta‐, and hexanucleotide) were not found in the three plastomes of *Toona*. Most SSRs were located in intergenic regions (72.5%), with few in introns (12.5%) and genes (15%). Overall, nine SSRs were shared by all three *Toona* species, including four in intergenic regions (*trnE‐UCC/trnT‐GGU*,* trnT‐GGU/psbD*,* ccsA/ndhD*, and *ycf15/rps12*), three in exons (*rpoC2*,* rpoB*, and *psbF*), and two in introns (*trnL‐UAA* and *ndhB*). In total, 23 repeats were detected in three *Toona* plastomes. A majority of the repeats (69.56%) were 30 to 40 bp in length, and 17.40% of the repeats were longer than 50 bp. Four repeats were shared by three *Toona* plastomes (Appendix S2). Additionally, we detected 466 SNPs (0.4%) and 90 indels among three plastomes, and we screened out four noncoding regions (*psbZ‐trnG*,* psbA‐trnK*,* trnF‐ndhJ*,* trnK‐rps16*) with potential to be loci for identification of *Toona* species (Appendix S3).

## DISCUSSION

In most angiosperm plastomes, the IR/LSC boundary lies within the *rps19* gene and the SSC/IR boundary lies within the *ycf1* gene (Kumar et al., 2009). Among the 29 Sapindales plastomes, the LSC/IR_B_ boundary of the majority lies within the *rps19* gene, while nine of these 29 plastomes have experienced an IR region expansion. Obvious IR region expansion to the LSC region has been detected in many other taxa, e.g., in *Pelargonium* L'Hér. (Chumley et al., 2006), *Tetracentron* Oliv. (Sun et al., 2013), and *Veronica nakaiana* Ohwi (Choi et al., 2016). In contrast, within Sapindales, there have been at least eight cases where the SSC/IR_A_ boundary has contracted to exclude all of *ycf1* (Fig. 4). IR region contraction has been found to occur in several ways, ranging from complete IR loss (e.g., Geraniaceae [Blazier et al., 2011], *Cephalotaxus oliveri* Mast. [Yi et al., 2013], and *Agathis dammara* (Lamb.) Rich. & A. Rich. [Wu and Chaw, 2014]), to the loss of tRNA genes within the IR region (e.g., *Epifagus virginiana* (L.) W. P. C. Barton [Morden et al., 1991] and *Bergera koenigii* L. [Shivakumar et al., 2016]), to the *rpl22* loss in rosids (Jansen et al., 2011), and to contraction at the IR/SSC boundaries reported in a number of early‐diverging angiosperms (e.g., *Buxus* L., *Epimedium* L., and *Macadamia* F. Muell.) (Hansen et al., 2007). Notably, in Rutaceae, all Clauseneae genera are characterized by the absence of *trnI‐GAU* and *trnA‐UGC* in the IR region. Tsuji et al. (2007) indicated that the tRNA loss may be caused by the RNA editing during the tRNA mutation. Pseudogenization of the *infA* gene has been detected in a number of angiosperm plastomes such as tobacco (Shinozaki et al., 1986), *Arabidopsis* Heynh. (Sato et al., 1999), and *Oenothera elata* Kunth (Hupfer et al., 2000), whereas among 29 Sapindales plastomes this was only detected in four plastomes (*Boswellia sacra*,* Acer davidii*,* A. morrisonense*, and *A. miaotaiense*) of Sapindaceae (Blazier et al., 2016). In some cases, the effect of plastid‐to‐nucleus gene transfer has been demonstrated to generate the pseudogenization of this gene (Millen et al., 2001).

As has been found in many other studies involving plastome‐scale phylogenetic analysis (Parks et al., 2009), we recovered improved phylogenetic support along the backbone of Sapindales compared to previous targeted gene analyses. We recovered Meliaceae as sister to the clade formed by Simaroubaceae (only one species included) + Rutaceae with maximal support, differing from the topology recovered by Muellner et al. (2007) and Muellner‐Riehl et al. (2016), where a moderately supported clade of Meliaceae + Simaroubaceae was sister to Rutaceae. Our result is consistent with the earlier work of Gadek et al. (1996) based on *trnL‐F* sequences, although they recovered only weak support. Unfortunately, the problem of the previously unsupported relationship of Sapindaceae with other Sapindales (Muellner‐Riehl et al., 2016) could also not be resolved by our plastome data analysis. It is important to emphasize caution for these results, however. Additional taxon sampling for complete plastomes, including additional lineages of already‐sampled families as well as the inclusion of the early‐diverging Sapindales families Biebersteiniaceae, Kirkiaceae, and Nitrariaceae may affect topology and support. Likewise, the plastome itself can be treated as a single locus for the purpose of phylogenetics, and genomic‐scale nuclear data may provide different estimates of phylogeny, especially for short branches.

Within Rutaceae, our results are highly congruent with those of the previous study (Shivakumar et al., 2016), which also found a clade of *Citrus* + *Merrillia* sister to a clade composed of (*Micromelum* + *Glycosmis*) + (*Murraya + Clausena*), although in the latter clade the bootstrap support was low. In our tree, all of the taxa sampled in Shivakumar et al. (2016) formed a clade, which is sister to *Zanthoxylum*. Our analysis suggests that tribe Clauseneae sensu Swingle and Reece (1967; *Micromelum* Blume, *Glycosmis* Corrêa, *Clausena* Burm. f., *Murraya* J. Koenig, and *Merrillia* Swingle) is not monophyletic because *Merrillia* is sister to *Citrus* L. of the tribe Citreae. The genera of Clauseneae are characterized by the absence of two tRNA genes (*trnI‐GAU* and *trnA‐UGC*), while this is not found in the genus *Citrus* (Fig. 4). Additionally, four genera (*Micromelum* + *Glycosmis* + *Murraya + Clausena*) in Rutaceae and two species (*Acer davidii* + *Acer morrisonense*) in Sapindales, characterized by the absence of *ycf1* in the SSC region, each formed a clade in our phylogenetic tree (Fig. 4). This gene loss shared by multiple taxa shows a particularly strong case of homoplasy in the phylogeny. Within Sapindaceae, *Sapindus* L. is sister to a clade containing *Dipteronia* Oliv. and *Acer* L. Although the support value is weak (57%), the two species of *Dipteronia* do not form a clade, instead forming a grade with respect to *Acer*.

The plastome structure and gene content of *Toona* reported in the present study enrich the available plastome resources within Sapindales, the comparative analyses among 29 plastomes provide insight into the plastome evolution of Sapindales, and the phylogenomic analyses of Sapindales improve our understanding of phylogenetic relationships within this order. In addition, the SSRs detected in three *Toona* species could provide a basis for future plastome genetic studies in *Toona*, especially in the threatened species.

## Appendix 1 Plastome assembly comparison among three *Toona* (Meliaceae) species.

**Table:**

Species	Read length (bp)	No. of plastid reads	No. of plastid contigs	Total no. of bases in contigs	Average depth of coverage (×)	Percentage of reads mapping	Contigs N50	Maximum/minimum contig size (bp)
*T. sureni*	150	119,147	60	810,415	112.72	99.27	24,245	80,048/2049
*T. sinensis*	150	102,323	81	845,099	96.71	99.91	21,131	84,024/2015
*T. ciliata*	150	134,438	108	794,274	130.88	99.76	10,027	86,924/2043

## Appendix 2 Exon and intron lengths (in base pairs) of genes in the three *Toona* (Meliaceae) plastomes.^a^

**Table:**

Gene	Exon1	Intron1	Exon2	Intron2	Exon3
*trnK‐UUU*	28/28/28	—	36/36/36	—	—
*trnG‐UCC*	23/23/23	724/724/724	49/49/49	—	—
*trnL‐UAA*	36/36/36	530/530/530	49/49/49	—	—
*trnV‐UAC*	36/36/36	600/602/602	38/38/38	—	—
*trnI‐GAU*	41/41/41	956/956/954	33/33/33	—	—
*trnA‐UGC*	37/37/37	841/841/841	34/34/34	—	—
*atpF*	438/438/438	720/720/720	155/155/155	—	—
*ndhA*	536/536/536	1099/1098/1098	551/551/551	—	—
*ndhB*	756/756/756	682/682/682	775/775/775	—	—
*rpl2*	433/433/433	671/671/671	390/390/390	—	—
*rps12* b	113/113/113	—	25/25/25	537/537/537	231/231/231
*rpoC1*	1619/1619/1619	753/753/753	434/434/434	—	—
*clpP*	198/198/198	690/690/690	290/290/290	860/860/860	67/67/67
*ycf3*	152/152/152	791/789/789	227/227/227	628/628/628	728/728/728

Notes

a Values presented correspond to *T. sureni/T. sinensis/T. ciliata*, respectively.

b Intron 1 of *rps12* is not shown because *rps12* is trans‐spliced.

## Appendix 3 Distribution of simple sequence repeats in the plastomes of three *Toona* (Meliaceae) species.

**Table:**

Species/Base	Length (bp)	Position in plastid genome
*Toona ciliata*		
A	10	9258–9267, 34,518–34,527, 38,871–38,880, 54,296–54,305, 57,729–57,738, 62,577–62,586, 67,493–67,502, 68,780–68,789, 74,450–74,459, 116,277–116,286, 118,064–118,073, 118,119–118,128, 134,208–134,217
	11	50,135–50,145, 73,952–73,962, 84,456–84,466, 116,647–116,657, 13,576–13,586, 147,115–147,125
	12	7044–7055, 31,827–31,838, 113,763–113,774
	13	143,677–143,689
	15	4738–4752
	16	78,707–78,722
T	10	14,085–14,094, 27,614–27,623, 70,109–70,118, 73,655–73,664, 73,788–73,797, 83,409–83,418, 83,918–83,927, 119,623–119,632, 128,850–128,859, 130,504–130,513, 131,949–131,958
	11	5882–5892, 7033–7043, 12,659–12,669, 31,412–31,422, 45,602–45,612, 54,222–54,232, 57,155–57,165, 57,236–57,246, 62,239–62,249, 63,742–63,752, 70,526–70,536, 99,025–99,035, 125,450–125,460
	12	9479–9490, 19,766–19,777, 52,802–52,813, 125,141–125,152, 132,376–132,387
	13	6828–6840, 74,806–74,818, 102,461–102,473
	15	48,905–48,919, 118,464–118,478
AT	10	21,267–21,276, 33,424–33,433, 49,396–49,405, 49,786–49,795, 50,733–50,742, 54,811–54,820, 121,130–121,139
*Toona sureni*		
A	10	34,799–34,808, 54,560–54,569, 57,999–58,008, 67,156–67,165, 67,798–67,807, 74,266–74,275, 116,568–116,577, 134,590–134,599
	11	13,859–13,869, 50,428–50,438, 62,866–62,876, 74,770–74,780, 118,360–118,370, 147,491–147,501
	12	7220–7231, 84,776–84,787, 118,408–118,419
	13	79,023–79,035, 144,054–144,046
	14	9769–9782
	17	4886–4902, 39,136–39,152
T	10	6989–6998, 9648–9657, 10,432–10,441, 12,954–12,963, 14,367–14,376, 27,928–27,937, 31,526–31,534, 32,502–32,511, 45,214–45,223, 54,487–54,496, 57,499–57,508, 60,086–60,095, 61,837–61,846, 62,536–62,545, 83,645–83,654, 112,278–112,287, 117,742–117,751, 119,996–120,005, 125,519–125,528, 129,225–129,234, 130,879–130,888, 132,330–132,339
	11	14,446–14,456, 57,418–57,428, 64,050–64,060, 74,061–74,071, 99,376–99,386, 120,092–120,102, 125,826–125,836
	12	20,080–20,091, 45,861–45,872, 70,828–70,839, 73,966–73,977, 85,407–85,418, 132,757–132,768
	13	31,711–31,723, 53,075–53,087, 75,127–75,139, 102,811–102,823
	16	49,165–49,180, 118,836–118,851
AT	10	21,581–21,590, 33,705–33,714, 49,669–49,678, 50,079–50,088, 55,074–55,083, 121,505–121,514
AG	12	29,112–29,123
*Toona sinensis*		
A	10	34,737–34,746, 39,096–39,105, 57,923–57,932, 62,771–62,780, 66,759–66,768, 67,688–67,697, 68,975–68,984, 74,149–74,158, 74,646–74,655, 116,472–116,481, 134,407–134,416
	11	13,706–13,716, 50,357–50,367, 84,652–84,662, 116,842–116,852, 147,314–147,324
	12	9384–9395, 32,046–32,057, 113,958–113,969
	13	143,876–143,878
	15	78,903–78,917
	18	4864–4881
T	10	14,215–14,224, 27,768–27,777, 31,065–31,074, 45,827–45,836, 49,496–49,505, 70,304–70,313, 73,985–73,994, 84,114–84,123, 112,128–112,137, 119,821–119,830, 127,827–127,836, 129,049–129,058, 130,703–130,712, 132,148–132,157
	11	6010–6020, 6956–6966, 31,631–31,641, 62,433–62,443, 63,936–63,946, 70,721–70,731, 73,851–73,861, 99,220–99,230, 125,648–125,658
	12	9607–9618, 12,788–12,799, 19,896–19,907, 53,002–53,013, 125,339–125,350, 132,575–132,586
	13	75,002–75,014, 49,128–49,141
	14	49,128–49,141
AT	10	33,644–33,653
TA	10	49,617–49,626, 50,954–50,963
