# Supplementary material for: Complete plastome sequencing from Toona (Meliaceae) and phylogenomic analyses within Sapindales
Source: Appl Plant Sci. 2018 Apr 27;6(4):e1040. doi: 10.1002/aps3.1040 (PMC5947613; doi:10.1002/aps3.1040)
Supplement: Supplementary file 2 [file APS3-6-e1040-s002.docx]

APPENDIX S2. The repeat sequences in three *Toona* plastomes.

*Toona sureni* plastome

| Repeat size | Type | Start position of first repeat | Start position of repeats found in other regions | Location | Region |
| --- | --- | --- | --- | --- | --- |
| 30 | F | 95,008 | 95,026 | IGS (*ycf2-trnL-CAA*) | IR |
| 32 | F | 111,504 | 111,569 | IGS (*rrn4.5-rrn5*) | IR |
| 30 | P | 80,741 | 80,741 | IGS (*petB-petD*) | LSC |
| 36 | P | 102,402 | 124,822 | IGS (*rps12-trnV-GAC*), *ndhA* intron | IR, SSC |
| 37 | F | 87,185 | 97,259 | IGS (*rps3-rpl22*), IGS (*ycf2- trnL-CAA*) | LSC, IR |
| 38 | F | 107,905 | 107,976 | IGS (*trnA-UGC-rrn23*) | IR |
| 38 | F | 87,184 | 87,221 | IGS (*rps3-rpl22*) | IR, LSC |
| 48 | F | 31,415 | 31,415 | IGS (*petN-psbM*) | LSC |
| 58 | P | 569 | 569 | IGS (*trnH-GUG*-*psbA*) | LSC |

*Note:* F = forward; IR = inverted repeat; LSC = large single copy; P = palindrome; R = reverse; SSC = small single copy.

*Toona sinensis* plastome

| Repeat size | Type | Start position of first repeat | Start position of repeats found in other regions | Location | Region |
| --- | --- | --- | --- | --- | --- |
| 30 | F | 94,852 | 94870 | IGS (*ycf2-trnL-CAA*) | IR |
| 32 | F | 111,149 | 111191 | IGS (*rrn4.5- rrn5*) | IR |
| 33 | F | 29,347 | 29380, 29449 | IGS (*rpoB- trnC-GCA*) | LSC |
| 36 | F | 102,247 | 124645 | IGS (*rps12-trnV-GAC*), *ndhA* intron | IR,SSC |
| 38 | F | 107,755 | 107829 | IGS (*trnA-UGC-rrn23*) | IR |
| 48 | P | 31,336 | 31336 | IGS (*rps12-trnV-GAC*), *ndhA* intron | IR,SSC |
| 58 | P | 550 | 550 | IGS (*trnH-GUG-psbA*) | LSC |
| 74 | F | 29,332 | 29396 | IGS (*rpoB- trnC-GCA*) | LSC |

*Note:* F = forward; IR = inverted repeat; LSC = large single copy; P = palindrome; R = reverse; SSC = small single copy.

*Toona ciliata* plastome

| Repeat size | Type | Start position of first repeat | Start position of repeats found in other regions | Location | Region |
| --- | --- | --- | --- | --- | --- |
| 30 | F | 94,657 | 94675 | IGS (*rps12-trnV-GAC*), *ndhA* intron | IR, SSC |
| 32 | F | 111,159 | 111224 | IGS (*rrn4.5- rrn5*) | IR |
| 36 | F | 102,052 | 124447 | IGS (*rps12-trnV-GAC*), *ndhA* intron | IR, SSC |
| 38 | F | 107,560 | 107634 | IGS (*trnA-UGC-rrn23*) | IR |
| 48 | P | 31,117 | 31117 | IGS (*petN-psbM*) | LSC |
| 58 | P | 420 | 420 | IGS (*trnH-GUG-psbA*) | LSC |

*Note:* F = forward; IR = inverted repeat; LSC = large single copy; P = palindrome; R = reverse; SSC = small single copy.
