# Supplementary material for: Complete plastome sequencing from Toona (Meliaceae) and phylogenomic analyses within Sapindales
Source: Appl Plant Sci. 2018 Apr 27;6(4):e1040. doi: 10.1002/aps3.1040 (PMC5947613; doi:10.1002/aps3.1040)
Supplement: Supplementary file 3 [file APS3-6-e1040-s003.docx]

APPENDIX S3. Summary of SNPs found among three *Toona* plastomes.

| Region | Divergence proportion (%) | SNP | Indels |
| --- | --- | --- | --- |
| *trnH-psbA* | 0.01113 | 5 | 3 |
| *psbA* | 0.00192 | 2 | 0 |
| *psbA-trnK* | 0.03758 | 1 | 0 |
| *matK* | 0.00327 | 5 | 0 |
| *trnK* intron | 0.00562 | 14 | 0 |
| *trnK-rps16* | 0.1416 | 11 | 2 |
| *rps16* | 0.0426 | 11 | 0 |
| *rps16* intron | 0.0534 | 46 | 0 |
| *rps16-trnQ* | 0.0107 | 27 | 0 |
| *trnQ-psbK* | 0.0085 | 3 | 0 |
| *psbK-psbI* | 0.0027 | 1 | 0 |
| *trnS-trnG* | 0.0045 | 7 | 8 |
| *trnG* intron | 0.0041 | 3 | 1 |
| *atpA* | 0.0007 | 1 | 0 |
| *atpF* | 0.0024 | 1 | 1 |
| *atpF* intron | 0 | 0 | 2 |
| *atpF-atpH* | 0 | 0 | 3 |
| *atpH-atpI* | 0.0066 | 8 | 1 |
| *atpI* | 0.0013 | 1 | 0 |
| *rps2-rpoC2* | 0.00491 | 1 | 0 |
| *rpoC2* | 0.0019 | 8 | 2 |
| *rpoC1* | 0.0015 | 38 | 0 |
| *rpoC1* intron | 0.00216 | 2 | 1 |
| *rpoB* | 0.0019 | 6 | 0 |
| *rpoB-trnC* | 0.00366 | 4 | 5 |
| *trnC-petN* | 0.00086 | 1 | 0 |
| *petN-psbM* | 0.00116 | 2 | 0 |
| *psbM-trnD* | 0.00125 | 1 | 0 |
| *trnE-trnT* | 0.00389 | 5 | 0 |
| *trnM-psbD* | 0.00089 | 3 | 0 |
| *psbD* | 0.00126 | 2 | 0 |
| *psbC* | 0.00047 | 1 | 0 |
| *psbC-trnS* | 0.00279 | 14 | 0 |
| *trnS-psbZ* | 0.01341 | 23 | 0 |
| *psbZ* | 0.01186 | 0 | 0 |
| *psbZ-trnG* | 0.02554 | 14 | 0 |
| *trnG-trnM* | 0 | 20 | 0 |
| *rps14* | 0.0022 | 1 | 0 |
| *psaB* | 0.0006 | 2 | 0 |
| *psaA* | 0.00089 | 3 | 0 |
| *psaA-ycf3* | 0.00635 | 6 | 0 |
| *ycf3* | 0.00264 | 8 | 2 |
| *ycf3* intron 1 | 0.00349 | 4 | 2 |
| *ycf3* intron 2 | 0 | 0 | 2 |
| *ycf3-trnS* | 0.00076 | 1 | 0 |
| *trnT-trnL* | 0.00276 | 4 | 3 |
| *trnL intron* | 0.00251 | 2 | 0 |
| *trnL-trnF* | 0.00179 | 1 | 0 |
| *trnF-ndhJ* | 0.09696 | 2 | 2 |
| *ndhK* | 0.00292 | 3 | 0 |
| *ndhC* | 0.00184 | 1 | 0 |
| *ndhC-trnV* | 0 | 0 | 2 |
| *trnV* intron | 0.00111 | 1 | 0 |
| *trnV-trnM* | 0.00252 | 1 | 0 |
| *atpE* | 0.00332 | 2 | 0 |
| *atpB* | 0.00178 | 4 | 0 |
| *atpB-rbcL* | 0.00085 | 2 | 1 |
| *rbcL* | 0.00093 | 2 | 0 |
| *rbcL-accD* | 0 | 0 | 1 |
| *accD* | 0.00227 | 5 | 0 |
| *accD-psaI* | 0.002 | 2 | 1 |
| *psaI-ycf4* | 0.00068 | 2 | 4 |
| *ycf4-cemA* | 0.00147 | 2 | 3 |
| *cemA* | 0.00477 | 5 | 0 |
| *petA* | 0 | 0 | 0 |
| *petA-psbJ* | 0.00624 | 11 | 1 |
| *psbE* | 0 | 0 | 0 |
| *psbE-petL* | 0.0026 | 5 | 0 |
| *petL-petG* | 0 | 0 | 0 |
| *trnP-psaJ* | 0 | 0 | 0 |
| *psaJ-rpl33* | 0.00304 | 2 | 1 |
| *rpl33* | 0.00333 | 1 | 0 |
| *rps18-rpl20* | 0.00109 | 1 | 0 |
| *rpl20* | 0.00189 | 1 | 0 |
| *rpl20-rps12* | 0.00091 | 1 | 0 |
| *clpP* | 0.00096 | 3 | 0 |
| *clpP* intron1 | 0.00105 | 1 | 5 |
| *clpP* intron2 | 0.00156 | 2 | 1 |
| *psbB-psbT* | 0.00358 | 1 | 0 |
| *psbH* | 0.003 | 1 | 1 |
| *petB* | 0 | 0 | 1 |
| *petD* | 0.00127 | 1 | 0 |
| *petD-rpoA* | 0 | 0 | 1 |
| *rpoA* | 0.00271 | 4 | 0 |
| *infA-rps8* | 0 | 0 | 2 |
| *rps8* | 0.00506 | 3 | 0 |
| *rpl14* | 0.00181 | 1 | 0 |
| *rpl16* | 0.00333 | 2 | 0 |
| *rpl16-rps3* | 0 | 0 | 5 |
| *rps3* | 0.00101 | 1 | 0 |
| *rpl22* | 0 | 0 | 1 |
| *rpl2* | 0 | 0 | 1 |
| *ycf2* | 0.00019 | 2 | 0 |
| *rps12-trnV* | 0.00037 | 1 | 1 |
| *trnI* intron | 0.0007 | 1 | 0 |
| *trnA* intron | 0.00079 | 1 | 0 |
| *ycf1-ndhF* | 0 | 0 | 1 |
| *ycf1* | 0.00221 | 7 | 1 |
| *ndhF-rpl32* | 0.01024 | 15 | 4 |
| *rpl32-trnL* | 0.00247 | 4 | 4 |
| *ccsA* | 0.00686 | 9 | 0 |
| *ccsA-ndhD* | 0 | 0 | 5 |
| *ndhD* | 0.00306 | 7 | 0 |
| *ndhG* | 0.00372 | 3 | 0 |
| *ndhI* | 0.00529 | 4 | 0 |
| *ndhA* | 0.00153 | 5 | 0 |
| *ndhA* intron | 0.00123 | 2 | 1 |
| *ndhA-ndhH* | 0 | 0 | 1 |
| *ndhH* | 0.00113 | 2 | 0 |
| *rps15* | 0.00244 | 1 | 0 |
| *rps15-ycf1* | 0.00316 | 2 | 0 |
| Total | — | 466 | 90 |
